# Development of a subset of forelimb muscles and their attachment sites requires the ulnar-mammary syndrome gene *Tbx3*

**DOI:** 10.1242/dmm.025874

**Published:** 2016-11-01

**Authors:** Mary P. Colasanto, Shai Eyal, Payam Mohassel, Michael Bamshad, Carsten G. Bonnemann, Elazar Zelzer, Anne M. Moon, Gabrielle Kardon

**Affiliations:** 1 Department of Human Genetics, University of Utah, 15 North 2030 East, Salt Lake City, UT 84112, USA; 2 Department of Molecular Genetics, Weizmann Institute of Science, 234 Herzl Street, Rehovot 76100, Israel; 3 Neuromuscular and Neurogenetic Disorders of Childhood Section, National Institutes of Health, Building 35, Room 2A-116, MSC 3705, 35 Convent Drive, Bethesda, MD 20892-3705, USA; 4 University of Washington School of Medicine, Department of Pediatrics, Division of Genetic Medicine, 1959 NE Pacific Street HSB I-607-F, Seattle, WA 98195-7371, USA; 5 Weis Center for Research, Geisinger Clinic, 100 North Academy Avenue, Danville, PA 17822, USA

**Keywords:** Ulnar-mammary syndrome, UMS, Tbx3, Limb, Muscle, Bone

## Abstract

In the vertebrate limb over 40 muscles are arranged in a precise pattern of attachment via muscle connective tissue and tendon to bone and provide an extensive range of motion. How the development of somite-derived muscle is coordinated with the development of lateral plate-derived muscle connective tissue, tendon and bone to assemble a functional limb musculoskeletal system is a long-standing question. Mutations in the T-box transcription factor, *TBX3*, have previously been identified as the genetic cause of ulnar-mammary syndrome (UMS), characterized by distinctive defects in posterior forelimb bones. Using conditional mutagenesis in mice, we now show that TBX3 has a broader role in limb musculoskeletal development. TBX3 is not only required for development of posterior forelimb bones (ulna and digits 4 and 5), but also for a subset of posterior muscles (lateral triceps and brachialis) and their bone eminence attachment sites. TBX3 specification of origin and insertion sites appears to be tightly linked with whether these particular muscles develop and may represent a newly discovered mechanism for specification of anatomical muscles. Re-examination of an individual with UMS reveals similar previously unrecognized muscle and bone eminence defects and indicates a conserved role for TBX3 in regulating musculoskeletal development.

## Abstract (precis)

**Summary:** The ulnar-mammary syndrome (UMS) gene, *Tbx3*, is required for development of posterior forelimb bones, muscles and their attachment sites. This broadens the UMS phenotype and suggests a new muscle-specification model.

## INTRODUCTION

The vertebrate limb musculoskeletal system is essential for structural support and locomotion. It is composed of muscle, which is surrounded by muscle connective tissue, and attached via tendons to bone. Therefore, morphogenesis of a functional musculoskeleton requires coordinated development of these tissues. However, defects in limb development are common, affecting 1 in 500 live births (Giele et al., 2001). Determining how the complex pattern of muscles, tendons and bones develops is important for understanding normal limb musculoskeletal development and the etiology of congenital limb defects.

Ulnar-mammary syndrome (UMS, OMIM 181450) is a pleiotropic congenital disorder characterized by limb malformations as well as defects in mammary, apocrine, cardiac, dental and genital development (Bamshad et al., 1997; Schinzel, 1987). Individuals with UMS exhibit variability in limb phenotypes, ranging from complete absence of the forearm and hand, to posterior limb defects including absence of the ulna and/or loss of posterior digits, to digit 5 hypoplasia (Bamshad et al., 1999, 1997). Autosomal dominant mutations in *TBX3*, a member of the T-box family of transcription factors, have been identified as the cause of UMS (Bamshad et al., 1999, 1997). Insights into TBX3 function in normal and UMS limb development have come from mouse mutants. Unlike humans, mice heterozygous for *Tbx3* germline null mutations are phenotypically normal. However, homozygous deletion of *Tbx3* in mice leads to limb bone defects that largely phenocopy individuals with UMS, although the resulting embryonic lethality limited analysis of the limb phenotype (Davenport et al., 2003; Frank et al., 2013). Recent conditional deletion of *Tbx3* in the lateral plate-derived limb bud has revealed that TBX3 is required for normal levels of SHH signaling, cell proliferation and development of posterior bones (Emechebe et al., 2016). Although descriptions of UMS limb phenotypes have entirely focused on bone phenotypes, the abnormal surface anatomy of UMS individuals (indicative of underlying soft tissue defects; Bamshad et al., 1999) and aberrant locomotion of *Tbx3* mutant mice (Emechebe et al., 2016) suggests that TBX3 may play a broader role in musculoskeletal development than previously thought.

Studies of limb musculoskeletal development have largely concentrated on the bones, which provide the scaffold for the musculoskeletal system. In the limb, cartilage is the earliest specified tissue and derives from the lateral plate mesoderm (Pearse et al., 2007). SOX9+ pre-chondrocytes are specified from mesenchymal condensations within the limb and then differentiate into Collagen 2+ (COL2+) chondrocytes, which give rise to cartilage (Olsen et al., 2000). Many growth factors, signaling pathways and transcription factors regulate the formation and morphogenesis of the limb bones (Karsenty et al., 2009; Olsen et al., 2000; Zelzer and Olsen, 2003). The site of action of these proteins is generally within the chondrocytes, but recent work has shown that HOXA11 and D11, expressed in the outer perichondrium, non-cell-autonomously regulate development of the radius, ulna, tibia and fibula (Swinehart et al., 2013).

After the first wave of chondrogenesis, which gives rise to the primary limb skeleton, a second population of chondrocytes gives rise to ridges, or eminences, along the surface of the bones (Blitz et al., 2013, 2009). These structures provide stable points for muscle attachment. TGFβ signaling is required for the specification of SOX9+SCX+ eminence progenitors, and BMP4 is required for their differentiation into COL2+ chondrocytes (Blitz et al., 2009). Although loss-of-function of TGFβ and BMP4 demonstrate that these growth factors are necessary for specification and differentiation of all bone eminences, how particular eminences are specified is unknown.

The limb muscles arise from myogenic progenitors that migrate from the somites into the limbs (Hutcheson et al., 2009). These myogenic progenitors (that express either Pax3 or Pax7) become committed myoblasts expressing MYOD and/or MYF5, differentiate into myocytes, and then fuse into multinucleate myofibers expressing sarcomeric proteins, such as myosin heavy chain (Murphy and Kardon, 2011). As myofibers differentiate they are concurrently patterned into over 40 limb muscles, and each one of these anatomical muscles is unique in its size, shape, fiber orientation, and origin and insertions sites (Kardon, 1998). In mouse, the basic pattern of muscles is established by embryonic day (E)14.5 (Kardon et al., 2003).

Previous studies have shown that the pattern of muscles is extrinsically controlled by the lateral plate mesoderm (Grim and Wachtler, 1991; Jacob and Christ, 1980; Kardon et al., 2003). In particular, muscle connective tissue derived from the lateral plate appears to be crucial; TCF4+ (also known as TCF7L2) connective tissue fibroblasts form a pre-pattern that seems to control where myofibers differentiate and thus determine the basic pattern of limb muscles (Kardon et al., 2003). In addition, TCF4+ fibroblasts regulate muscle fiber type and the switch from fetal to adult myogenesis (Mathew et al., 2011). Genetic studies have established that several transcription factors expressed in lateral plate non-cell-autonomously regulate limb muscle morphogenesis. TBX5 and TBX4 regulate the general individuation of anatomical muscles in the fore- and hindlimbs, respectively (Hasson et al., 2010), whereas HOXA11 and D11 regulate individuation of muscles in the forearm (Swinehart et al., 2013). Additionally, LMX1B specifies the pattern of the distal dorsal muscles (Li et al., 2010). However, mechanisms controlling other aspects of muscle regional identity (e.g. anterior-posterior or proximal-distal) or the specification of particular individual muscles are unknown.

Morphogenesis of a functional musculoskeletal system requires the coordinated development of lateral plate-derived bone, muscle connective tissue and tendon with somite-derived muscle. The primary bones develop independently of muscle and tendon (Blitz et al., 2013, 2009; Kahn et al., 2009; Pryce et al., 2009). In contrast, initiation of bone eminences requires signals from tendon but not muscle, whereas subsequent growth and maintenance of eminences requires muscle contraction (Blitz et al., 2013, 2009). Tendons also initially develop independently of muscle, but their later development requires signals from muscle and/or cartilage depending on their location in the limb (Bonnin et al., 2005; Huang et al., 2015, 2013; Kardon, 1998; Schweitzer et al., 2001). Finally, the muscle connective tissue develops independently of muscle and appears to be crucial for regulating muscle morphogenesis (Hasson et al., 2010; Kardon et al., 2003; Swinehart et al., 2013). Thus, although it is clear that development of the musculoskeletal system involves tissue interdependencies, many of the cellular and molecular interactions between musculoskeletal components have yet to be elucidated.

Here, we show that TBX3 is important for development of three components of the limb musculoskeleton; primary bone, bone eminences and muscle. We find that *Tbx3* is specifically expressed in the precursors of posterior forelimb bones (ulna and digits 4 and 5) and, as shown previously (Emechebe et al., 2016; Frank et al., 2013), is required for their development. In addition, TBX3 cell-autonomously regulates the development of a subset of bone eminences (greater tubercle of the humerus, deltoid tuberosity and olecranon). Surprisingly, TBX3 also non-cell-autonomously regulates differentiation of two specific muscles (lateral triceps and brachialis) in the forelimb. The localization of muscle and bone eminence defects along the posterior side of the dorsal-ventral border of the upper forelimb of *Tbx3* mutants demonstrates that TBX3 is crucial for regulating musculoskeletal development of this region. Examination of an individual with UMS reveals similar previously unrecognized muscle and bone eminence defects and indicates a conserved role for TBX3 in musculoskeletal development.

## RESULTS

### Cells in the TBX3 lineage are found in a subset of forelimb bones, tendons and muscle connective tissue

**Fig. 1.**
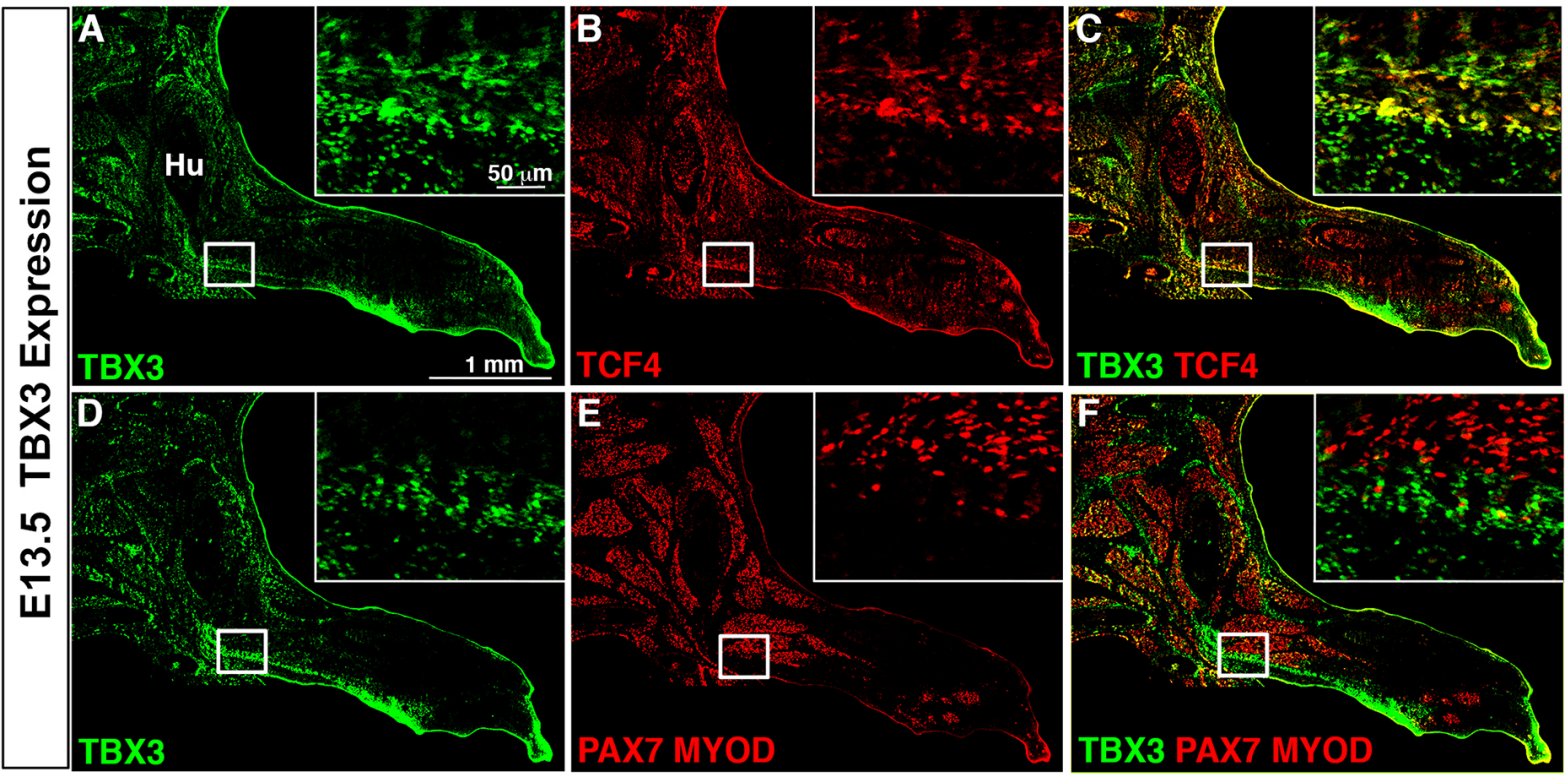
**TBX3 protein is expressed in the anterior and posterior forelimb.** (A,D) TBX3 is expressed in the anterior margin of the proximal forelimb and posterior margin along the length of the forelimb at E13.5. (A-F) TBX3 is expressed in TCF4+ connective tissue fibroblasts (A-C), but not in PAX7+ MYOD+ myogenic cells (D-F). Section immunofluorescence with (A-C) or without (D-F) antigen retrieval. White boxes indicate enlarged areas. Hu, humerus.

To determine in which regions and cell types TBX3 is expressed in the developing forelimb, we analyzed TBX3 by immunofluorescence in mouse at E13.5. Consistent with previous studies (Gibson-Brown et al., 1996), we find that TBX3 is expressed in the anterior and posterior margins of the forelimb (Fig. 1A,D). In these regions, TBX3 is co-localized with a subset of lateral plate-derived cells such as TCF4+ muscle connective tissue fibroblasts (Fig. 1A-C). However, TBX3 does not co-localize with somite-derived PAX7+ muscle progenitors or MYOD+ myoblasts (Fig. 1D-F).

**Fig. 2.**
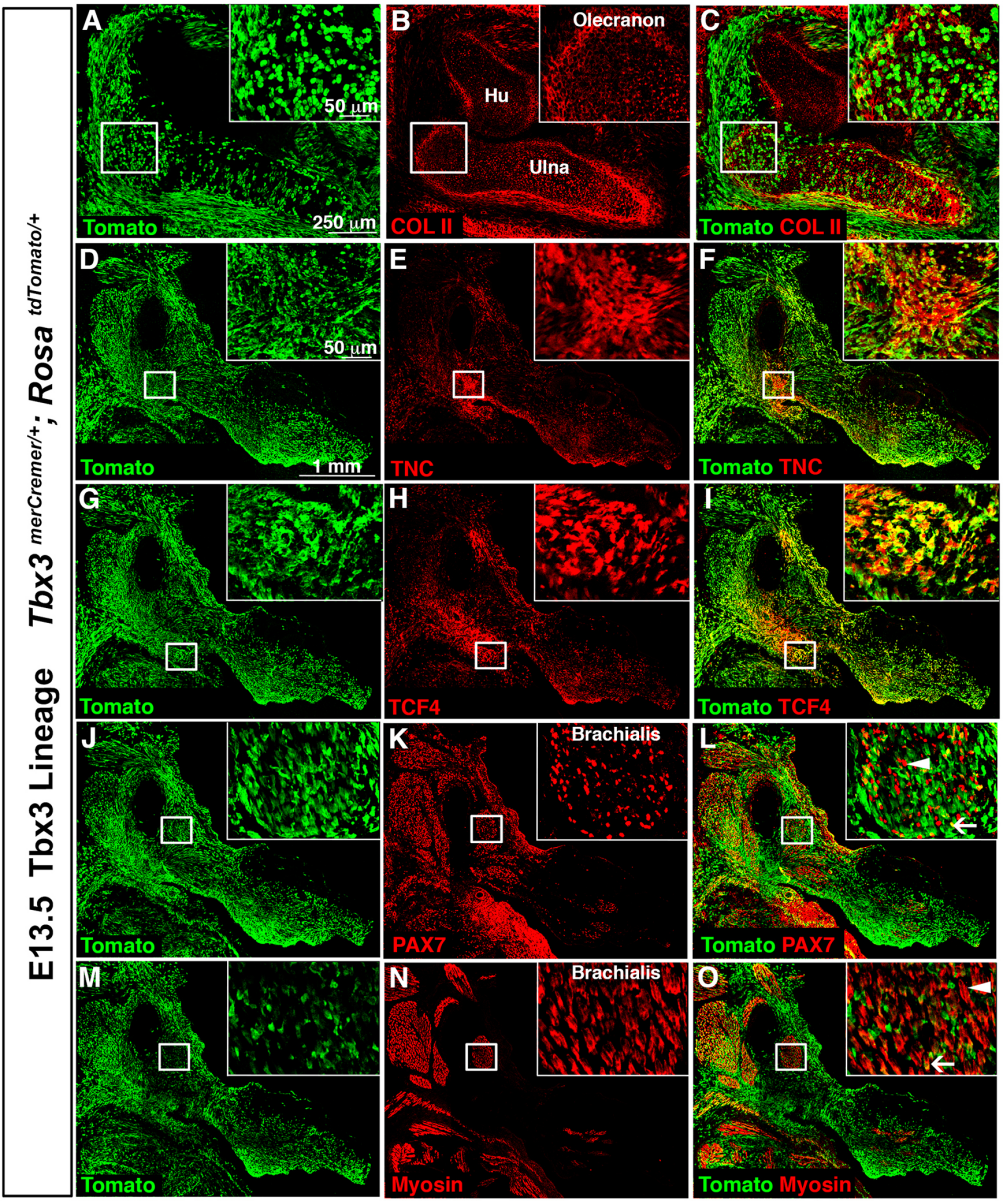
**Cells in the Tbx3 lineage are found in a subset of forelimb bones, tendons and muscle connective tissue.** *Tbx3+* cells, labeled by means of tamoxifen administered to E9.5 *Tbx3^merCremer/+^; Rosa^tdTomato/+^* embryos and analyzed at E13.5, are found in posterior bones (ulna, digits 3-5), a subset of bone eminences (e.g. olecranon) (A-C), Tenascin C+ (TNC+) tenocytes (D-F) and TCF4+ connective tissue fibroblasts (G-I). Most PAX7+ myogenic cells do not derive from *Tbx3+* cells (L, arrowhead), but a few PAX7+ cells derive from *Tbx3*+ cells (L, arrow). *Tbx3*+ cells generally do not give rise to Myosin+ myofibers (O, arrowhead), but some myofibers derive from *Tbx3*+ cells (O, arrow). Section immunofluorescence. White boxes indicate enlarged areas. Hu, humerus.

To determine more definitively which cell types derive from *Tbx3*+ cells, we utilized *Tbx3^merCremer/+^; Rosa^tdTomato/+^* embryos (Emechebe et al., 2016) in which *Tbx3*+ cells were genetically labeled at E9.5 (by means of tamoxifen delivered to pregnant dams) as Tomato+ and then analyzed at E13.5 (Fig. 2). Chondrocytes derived from *Tbx3*+ cells are found in the posterior primary bones (ulna, digits 4 and 5, and posterior part of digit 3) as well as bone eminences, such as the olecranon (Fig. 2A-C). In addition, Tenascin C+ (TNC+) tenocytes in the posterior limb and the proximal, anterior forelimb (Fig. 2D-F) and TCF4+ fibroblasts associated with multiple muscles are derived from *Tbx3*+ cells (Fig. 2G-I). In muscle, most PAX7+ myogenic progenitors and Myosin+ myofibers are not Tomato+ and so not derived from *Tbx3*+ cells (Fig. 2J-L), although in a few muscles some myofibers are Tomato+ (Fig. 2M-O). Thus, in the developing forelimb, the *Tbx3* lineage is primarily found in lateral plate-derived cells in posterior bones and a subset of bone eminences, tendon and muscle connective tissue.

### TBX3 is required for development of a subset of bones and bone eminences

**Fig. 3.**
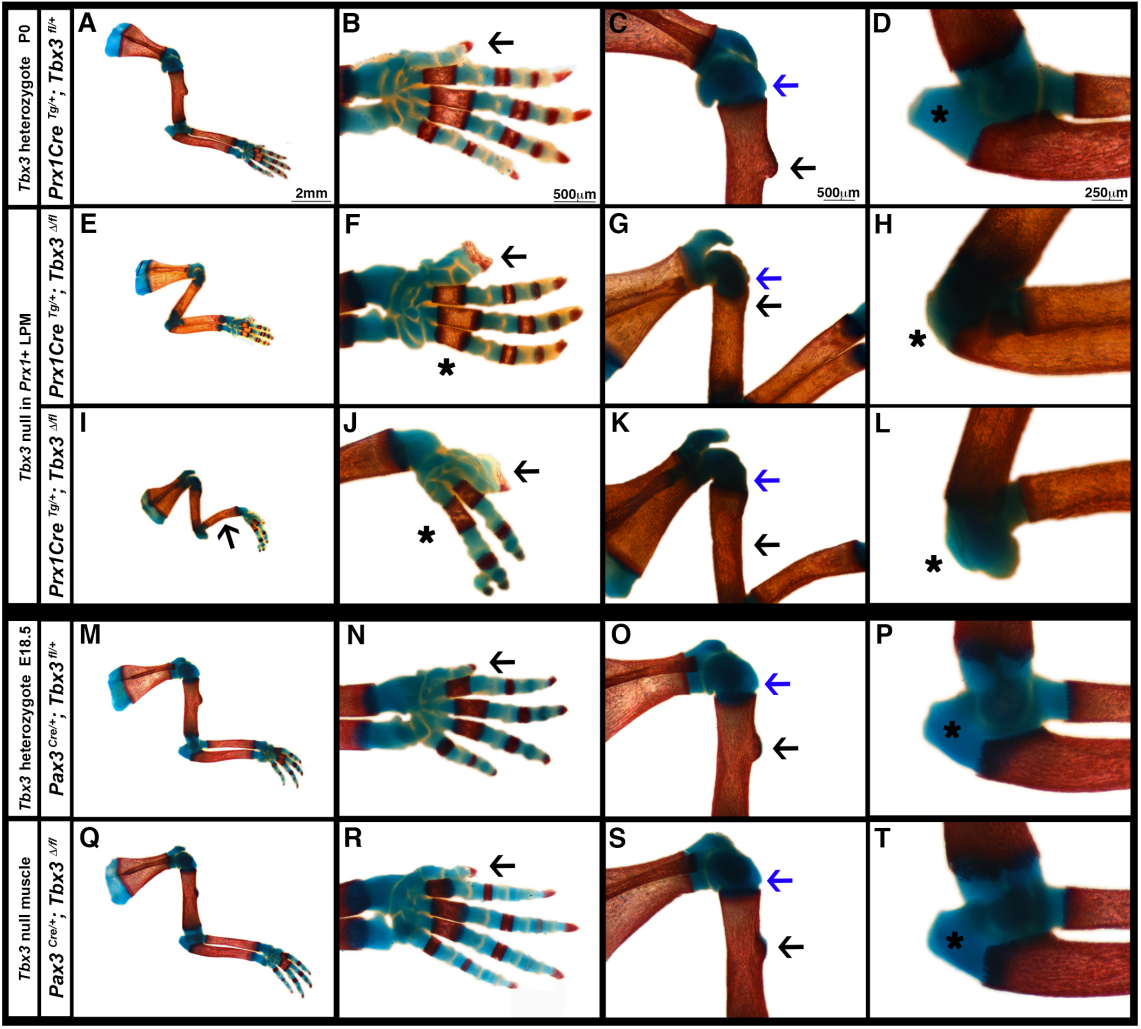
**TBX3 is required for development of a subset of bones and bone eminences.** At P0, homozygous deletion of *Tbx3* in *Prx1*+ cells results in shorter humerus, radius and ulna (E,I, *n*=27/27), duplication of the first digit (F,J, arrow, *n*=27/27), loss of posterior digits (F,J, asterisk, *n*=19/27), absence of the greater tubercle (G,K, blue arrow, *n*=8/10) and deltoid tuberosity (G,K, black arrow, *n*=10/10), and absent or truncated olecranon (H,L, asterisk, *n*=10/10), and in some animals loss of the ulna (I, arrow, *n*=3/27). (M-T) No skeletal defects are present with deletion of one *Tbx3* allele in *Prx1*+ cells (A-D, *n*=16/16) or with heterozygous or homozygous *Tbx3* deletion in *Pax3*+ muscle progenitors (M-P, *n*=14/14; Q-T, *n*=8/8). Whole-mount Alcian Blue and Alizarin Red staining.

To determine the role of TBX3 in limb development and circumvent early embryonic lethality, we conditionally deleted *Tbx3* in the limb of mice using a floxed allele of *Tbx3* (Frank et al., 2012) and the *Prx1Cre* transgene (Logan et al., 2002). *Prx1Cre* is expressed throughout the forelimb lateral plate-derived mesoderm, which gives rise to the limb bones, tendons and muscle connective tissue by E9.5 (Fig. S1A-E) (Logan et al., 2002; Pearse et al., 2007). In response to Cre, the *Tbx3^fl^* allele deletes exon 1 and produces a functional null allele (Frank et al., 2012). At postnatal day (P)0, *Prx1Cre^Tg/+^*; *Tbx3^fl/+^* heterozygous animals exhibit no skeletal defects (Fig. 3A-D). However, in all *Prx1Cre^Tg/+^*; *Tbx3^Δ/fl^* mutants the forelimbs are shorter, the first digit is duplicated and posterior digits are absent (Fig. 3E,F; Table S1). In some mutants (*n*=3/27) the ulna is absent (Fig. 3I,J) (Emechebe et al., 2016). There is some variability in the earliest time of Cre-mediated deletion by the *Prx1Cre* transgene, and it is presumably in mice with the earliest Cre-mediated deletion of *Tbx3* (at E9.0 in embryos with 14 somites, data not shown) that the ulna is absent. With the exception of the duplicated first digit, these defects recapitulate those seen in individuals with UMS (Bamshad et al., 1999, 1997). To further test that TBX3 is specifically required in lateral plate-derived limb bud cells, we also deleted *Tbx3* in somite-derived cells using *Pax3^Cre^* (Engleka et al., 2005). However, neither *Pax3^Cre/+^*; *Tbx3^fl/+^* heterozygous controls nor *Pax3^Cre/+^*; *Tbx3^Δ/fl^* mutants showed any limb skeletal defects (Fig. 3M-T). Together, these data demonstrate that TBX3 functions in the lateral plate-derived limb bud mesoderm, and that *Prx1Cre^Tg/+^*; *Tbx3^Δ/fl^* mice largely recapitulate the range of bone defects found in UMS individuals.

In addition to the primary skeletal defects, *Prx1Cre^Tg/+^*; *Tbx3^Δ/fl^* mutants also exhibit defects in bone eminences. These mice are missing the greater tubercle of the humerus and deltoid tuberosity (Fig. 3G,K; Table S1) and have an absent or severely truncated olecranon (Fig. 3H,L; Table S1). However, not all eminences are affected; for instance the lateral epicondyle of the humerus develops normally in the absence of TBX3 (data not shown). Eminences found in regions where TBX3 is expressed (Fig. 1A and data not shown) develop abnormally in the absence of TBX3. Furthermore, the high levels of recombination by *Prx1Cre* in bone eminences (Fig. S1D,E) suggests that TBX3 is required cell-autonomously for development of this subset of eminences.

**Fig. 4.**
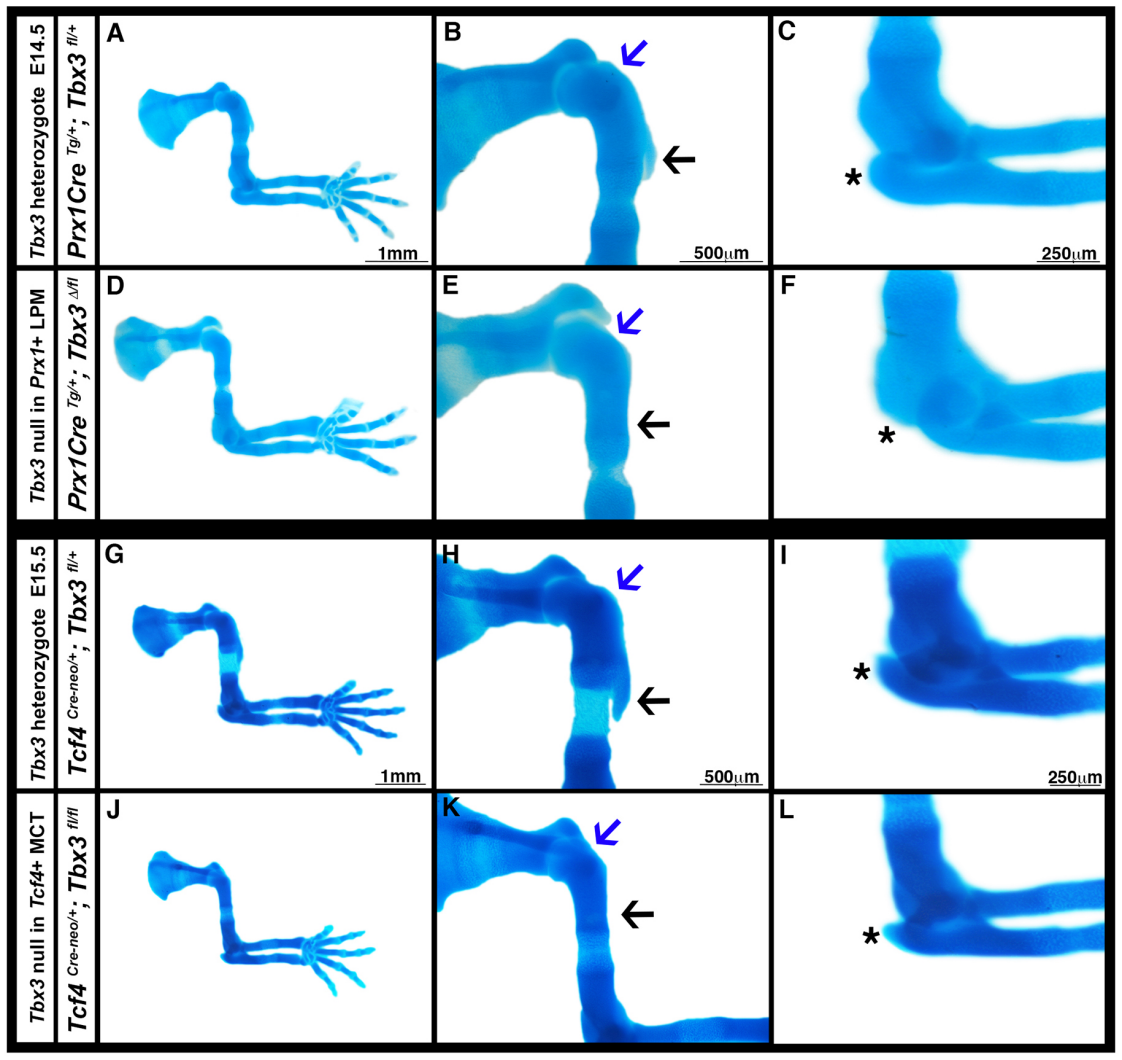
**TBX3 is required for the initiation of bone eminences.** (A-C) At E14.5 no skeletal defects are present with deletion of one *Tbx3* allele in *Prx1*+ cells (*n*=6/6). (D-F) Homozygous deletion of *Tbx3* in *Prx1*+ cells results in the absence of the greater tubercle (E, blue arrow), deltoid tuberosity (E, black arrow) and olecranon (F, asterisk) (*n*=8/8). (G-I) No skeletal defects are present with deletion of one *Tbx3* allele in *Tcf4*+ cells (*n*=2/2). (J-L) At E15.5 homozygous deletion of *Tbx3* in *Tcf4*+ cells results in absence of the greater tubercle (K, blue arrow) and deltoid tuberosity (K, black arrow) and a slightly smaller olecranon (L, asterisk) (*n*=4/4). Whole-mount Alcian Blue staining. Blue arrows, greater tubercle; black arrows, deltoid tuberosity; asterisks, olecranon.

Bone eminence development occurs in two phases. In the initial phase chondrocytes give rise to the nascent eminences, and in the later phase muscle contraction is required to maintain these structures (Blitz et al., 2009). To determine during which phase of secondary skeletal development TBX3 is required, we examined limb skeletons at E14.5, when the initial phase is complete. No defects were present in *Prx1Cre^Tg/+^*; *Tbx3^fl/+^* heterozygous controls (Fig. 4A-C), but in *Prx1Cre^Tg/+^*; *Tbx3^Δ/fl^* mutants the greater tubercle and the deltoid tuberosity are absent and the olecranon is truncated (Fig. 4D-F). Therefore, TBX3 is required for the initial development of a subset of bone eminences.

To further delimit when TBX3 is required for development of this subset of eminences, we analyzed *Tcf4^Cre-neo/+^*; *Tbx3^fl/fl^* embryos. *Tcf4^Cre-neo^* drives recombination later than *Prx1Cre*, beginning at E9.5, and is largely restricted to muscle connective fibroblasts and bone eminences (Fig. S1F-J) (Mathew et al., 2011). Whereas bone eminences are normal in *Tcf4^Cre-neo/+^*; *Tbx3^fl/+^* controls, in *Tcf4^Cre-neo/+^*; *Tbx3^fl/fl^* mutants the greater tubercle and deltoid tuberosity are absent, although the olecranon is only slightly smaller (Fig. 4G-L; Table S1). Therefore, because *Tcf4^Cre-neo^* recombines later than *Prx1Cre* in the eminences, this suggests that development of the greater tubercle and deltoid tuberosity requires TBX3 at E9.5 and beyond, whereas the olecranon requires TBX3 for a brief period, at ∼E9.0-9.25 (Table S1).

**Fig. 5.**
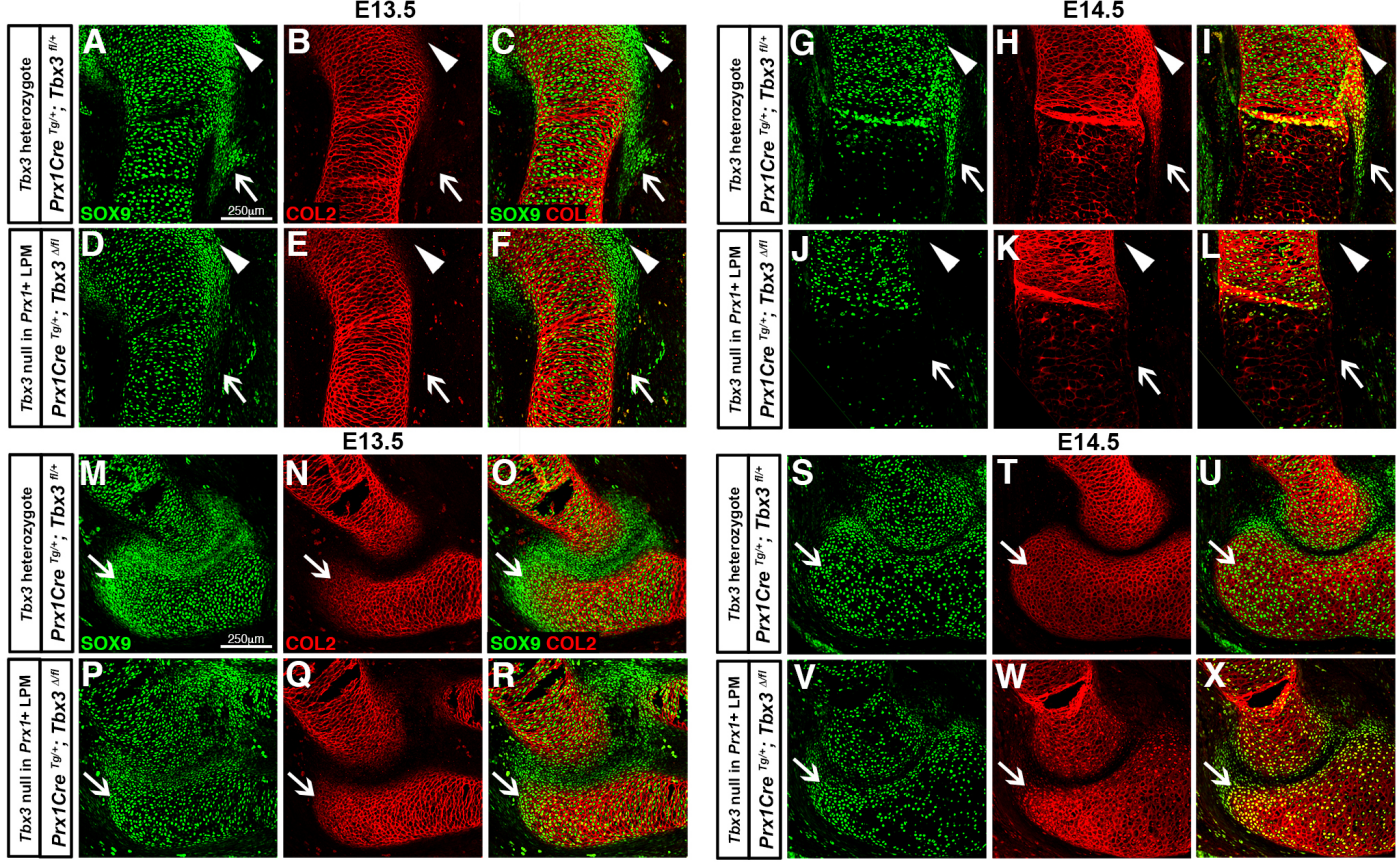
**TBX3 regulates development of the greater tubercle, deltoid tuberosity and olecranon.** (A-C) Cells that give rise to the greater tubercle (arrowheads) and deltoid tuberosity (arrows) are normally specified (SOX9+) but have not differentiated (COL2–) in E13.5 mice with deletion of one *Tbx3* allele in *Prx1*+ cells. (D-F) In embryos where *Tbx3* has been deleted in *Prx1*+ cells SOX9+ progenitors are present in the greater tubercle (arrowheads), but absent in the deltoid tuberosity (arrows) (*n*=6). (G-I) At E14.5 cells that give rise to the greater tubercle (arrowheads) and deltoid tuberosity (arrows) are normally specified (SOX9+) and differentiated (COL2+) in *Tbx3* heterozygotes. (J-L) When *Tbx3* has been deleted in *Prx1*+ cells, greater tubercle (arrowheads) and deltoid tuberosity (arrows) SOX9+ progenitors and differentiated COL2+ chondrocytes are absent (*n*=6). (M-O) At E13.5 cells that give rise to the olecranon (arrows) are normally specified (SOX9+) but have not differentiated (COL2–) in control *Tbx3* heterozygotes. (P-R) In mutants, olecranon SOX9+ chondrocyte progenitors are present in fewer numbers (arrows) (*n*=6). (S-U) At E14.5 cells that give rise to the olecranon (arrows) have normally differentiated (COL2+) in *Tbx3* heterozygotes. (V-X) In mutants, fewer SOX9+COL2+ differentiated chondrocytes contribute to a truncated olecranon (arrows) (*n*=6). Section immunofluorescence.

During the initial phase of bone eminence development, SOX9+ chondrocyte progenitors are specified and then differentiate into COL2+ chondrocytes (Blitz et al., 2013). To determine whether TBX3 is required for specification and/or differentiation of the greater tubercle, deltoid tuberosity and olecranon we used section immunofluorescence. At E13.5 in *Prx1Cre^Tg/+^*; *Tbx3^fl/+^* control embryos, SOX9+ progenitors that give rise to the greater tubercle, deltoid tuberosity and olecranon have been specified (Fig. 5A-C,M-O). In *Prx1Cre^Tg/+^*; *Tbx3^Δ/fl^* mutants, SOX9+ progenitors are present in the greater tubercle, absent from the deltoid tuberosity, and reduced in the olecranon (Fig. 5D-F,P-R). By E14.5 in *Prx1Cre^Tg/+^*; *Tbx3^fl/+^* controls most SOX9+ cells have differentiated and express COL2 in the three eminences (Fig. 5G-I,S-U). However, in *Prx1Cre^Tg/+^*; *Tbx3^Δ/fl^* mutants no COL2+ chondrocytes are present in the greater tubercle or deltoid tuberosity (Fig. 5J-L), and there are markedly fewer COL2+ chondrocytes in the olecranon (Fig. 5V-X). Thus, TBX3 regulates chondrocyte specification of the deltoid tuberosity and olecranon, and chondrocyte differentiation of the greater tubercle.

### TBX3 non-autonomously regulates development of a subset of forelimb muscles

**Fig. 6.**
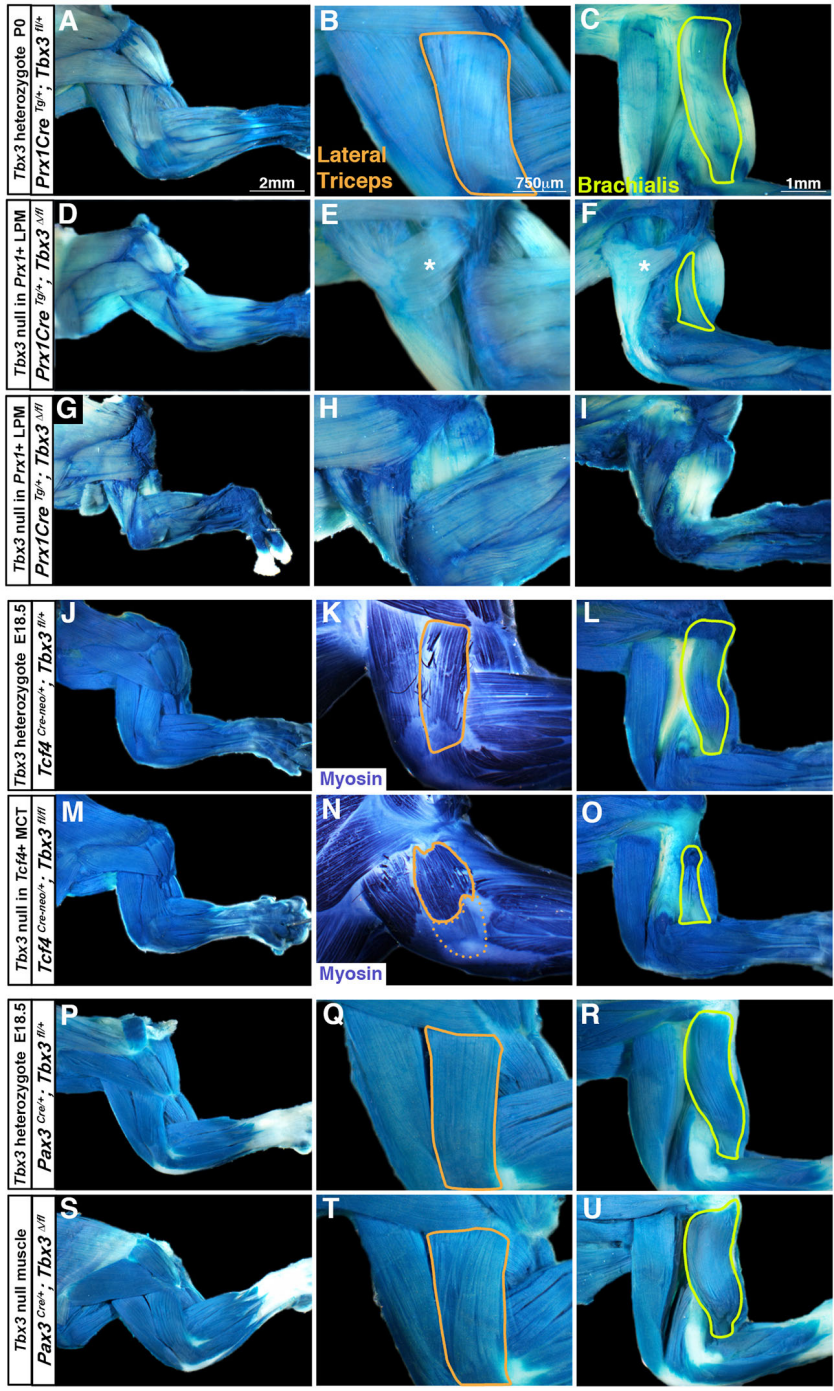
**TBX3 is required in lateral plate-derived limb mesoderm, but not in muscle, for development of a subset of forelimb muscles.** (A-C) All muscles develop normally, including lateral triceps (B, outlined in orange) and brachialis (C, outlined in yellow) in P0 mice with deletion of one *Tbx3* allele in *Prx1*+ lateral plate-derived limb mesoderm (*n*=8/8). (D-F) Deletion of *Tbx3* in *Prx1*+ cells results in smaller limbs, loss of the lateral triceps (E, outlined in orange in B), presence of a superficial, ectopic, non-functional muscle (E,F; asterisk), and a hypoplastic brachialis (F, outlined in yellow in C) (*n*=15/15). (G-I) In mutants where the ulna is absent both the lateral triceps (H) and brachialis (I) are absent (*n*=2/2). (J-L) No muscle-patterning defects are present in mice in which one *Tbx3* allele is deleted in *Tcf4*+ cells (*n*=2/2). (M-O) Deletion of *Tbx3* in *Tcf4*+ cells results in a hypoplastic lateral triceps (N) (*n*=2/2) and brachialis (O) (*n*=2/2). (P-U) No muscle patterning defects are present in mice in which one (*n*=5/5) or two (*n*=3/3) *Tbx3* alleles are deleted in *Pax3*+ muscle progenitors. Myofibers are β-galactosidase+ because *Tcf4^Cre-neo^* later drives recombination in myonuclei (Mathew et al., 2011). All mice include the *Rosa^LacZ/+^* allele and shown are whole-mount β-galactosidase staining of *Prx1*-derived (A-I), *Tcf4*-derived (J,L,M,O) or *Pax3*-derived (P-U) cells. (K,N) Whole-mount myosin-alkaline phosphatase staining. Lateral triceps has been removed in C,F,I,L,O,R,U.

Although muscle abnormalities have not previously been identified in UMS individuals, our analysis of the gait of adult *Prx1Cre^Tg/+^*; *Tbx3^Δ/fl^* mutant animals suggested muscle defects. Compared with control mice (Movie 1), mutant animals displayed an abnormal gait in which they have difficulty fully flexing or extending the forearm and paw (Movie 2). To determine whether *Tbx3* mutants have muscle defects, we analyzed P0 *Prx1Cre^Tg/+^*; *Tbx3^fl/+^*; *Rosa^Lacz/+^* control and *Prx1Cre^Tg/+^*; *Tbx3^Δ/fl^*; *Rosa^Lacz/+^* mutant mice, in which β-galactosidase+ muscle connective tissue allows for visualization of the muscles. Whereas the control mice had a normal muscle pattern (Fig. 6A-C), the mutants had defects in a specific subset of forelimb muscles (Fig. 6D-I; Table S1). In all mutants analyzed the lateral head of the triceps was absent bilaterally (Fig. 6E,H; normal lateral triceps outlined in B). In addition, the brachialis (that lies just medial to the lateral triceps) was also defective. In mutants with less severe bone phenotypes (reduced number of digits and duplicated thumb, but ulna present) on both right and left forelimbs the brachialis was smaller and its origin shifted distally from the greater tubercle to the anterior mid-shaft of the humerus (Fig. 6F; normal brachialis outlined in C). In these less­ severe mutants, an additional superficial ectopic muscle was present that originates from the superficial surface of the long head of the triceps and inserts on the distal head of the acromiodeltoideus. Because it does not cross a joint, this ectopic muscle cannot function to move the skeleton. In two of the rarer *Prx1Cre^Tg/+^*; *Tbx3^Δ/fl^* mutants in which the skeletal phenotype was more severe (the ulna was absent), in addition to the loss of the lateral triceps, the brachialis was missing and the ectopic muscle was variably present (Fig. 6G-I).

To further delimit the requirement for TBX3 in muscle development, we conditionally deleted *Tbx3* using the *Tcf4^Cre-neo^* allele, which drives recombination later than *Prx1Cre*, beginning at E9.5, and is largely restricted to muscle connective fibroblasts and bone eminences (Fig. S1F-J) (Mathew et al., 2011). At E18.5, forelimbs of *Tcf4^Cre-neo/+^*; *Tbx3^fl/fl^* mutants displayed a subset of defects seen in *Prx1Cre^Tg/+^*; *Tbx3^Δ/fl^* mutants. Similar to the less severe *Prx1Cre^Tg/+^*; *Tbx3^Δ/fl^* mutants, in *Tcf4^Cre-neo/+^*; *Tbx3^fl/fl^* mutants the brachialis is truncated proximally (Fig. 6O; Table S1). However, in *Tcf4^Cre-neo/+^*; *Tbx3^fl/fl^* mutants, the lateral triceps was present, although both the proximal and distal ends were truncated; this resulted in a shift of the origin distally along the humerus and a shift of the insertion proximally from the olecranon to the lateral, mid-shaft of the humerus (Fig. 6N; Table S1). Additionally, no ectopic muscle was present in mutants (Fig. 6N). Thus, the muscle phenotype is less severe when *Tbx3* is deleted via *Tcf4^Cre-neo^*.

Although we have shown that TBX3 in lateral plate-derived limb mesoderm regulates development of the lateral triceps and brachialis, TBX3 might also have a cell autonomous role in muscle development (as we found a few myofibers derived from *Tbx3*+ cells). To test this, we deleted *Tbx3* in limb muscle using *Pax3^Cre^*. In both *Pax3^Cre/+^*; *Tbx3^fl/+^* controls and *Pax3^Cre/+^*; *Tbx3^Δ/fl^* mutants we saw no muscle defects (Fig. 6P-U). Thus, TBX3 is not required in myogenic cells, but acts exclusively in lateral plate-derived cells to pattern a subset of forelimb muscles.

Together, these results show that TBX3 in the non-muscle limb mesoderm regulates a specific subset of neighboring forelimb muscles (Table S1). The lateral triceps and adjacent brachialis are the primary muscles affected by TBX3 loss (other minor defects in the nearby acromiodeltoideus are also present). Interestingly, the nature of these defects is strongly affected by the timing of *Tbx3* deletion. Early *Tbx3* deletion (at ∼E9.0, in severe *Prx1Cre^Tg/+^*; *Tbx3^Δ/fl^* mutants that are missing the early specified ulna as well as posterior digits) results in the loss of both the lateral triceps and the brachialis. Slightly later *Tbx3* deletion (at ∼E9.25, in less severe *Prx1Cre^Tg/+^*; *Tbx3^Δ/fl^* mutants in which ulnas are present, but the most posterior digit is either missing or hypoplastic) results in the loss of the lateral triceps and only a truncated brachialis. Later *Tbx3* deletion (at ∼E9.5, in *Tcf4^Cre-neo/+^*; *Tbx3^fl/fl^* mutants in which ulnas and all digits are present) results in only truncation of the lateral triceps and brachialis. Thus, development of the lateral triceps requires TBX3 in the lateral plate-derived limb mesoderm at ∼E9.0-9.5, whereas the brachialis requires TBX3 for a briefer period, at ∼E9.0-9.25.

Intriguingly, in the various *Tbx3* conditional mutants the phenotypes of the muscles are tightly correlated with the phenotypes of the bone eminences to which they attach (Table S1). The lateral triceps normally originates on the greater tubercle and inserts onto the olecranon, whereas the brachialis originates on the greater tubercle and attaches to the ulna. With early *Tbx3* deletion (∼E9.0), the greater tubercle, olecranon and ulna are absent, and the lateral triceps and brachialis that originate and insert on these eminences are absent. With slightly later *Tbx3* deletion (∼E9.25), the greater tubercle is absent and olecranon severely truncated, but the ulna is present; correspondingly the lateral triceps is absent, whereas the brachialis is present but truncated at its origin end. Finally, with later *Tbx3* deletion, the greater tubercle is absent, the olecranon is slightly smaller, and the ulna is present; correspondingly, the lateral triceps is present but truncated at its origin and insertion ends and the brachialis is present but truncated at its origin end. These two muscles are the only ones that span between and attach at these eminences and their phenotype perfectly correlates to the state of their attachments. This strongly suggests that development of these muscles is tightly linked to the development of their attachment sites.

### TBX3 is required for proper myofiber formation and orientation of a subset of forelimb muscles

**Fig. 7.**
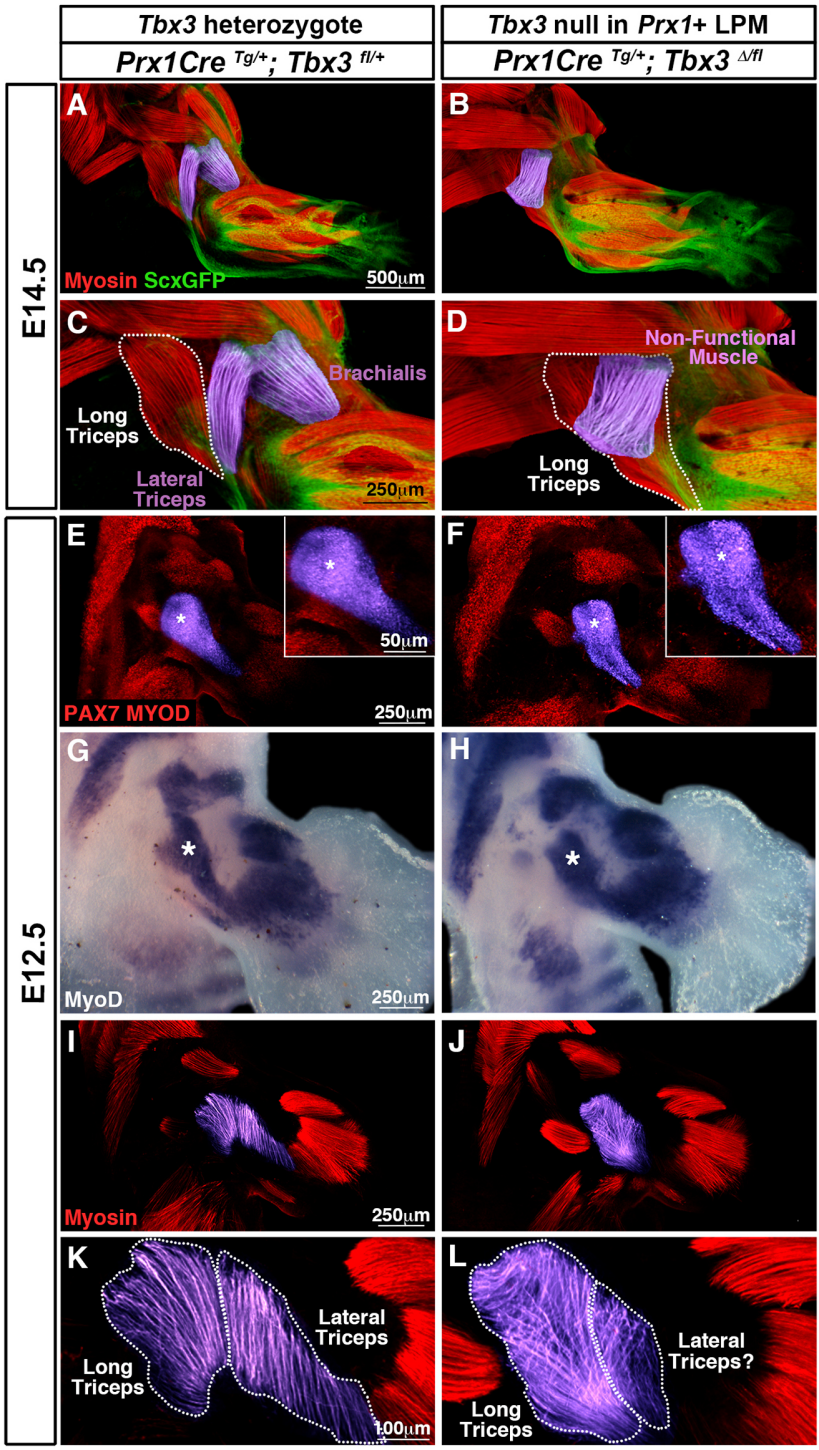
**TBX3 regulates myofiber formation and orientation in the forelimb during development.** (A,C) E14.5 normal muscle pattern, showing the lateral triceps and brachialis (pseudo-colored in purple) and the long triceps (outlined in white) with one allele of *Tbx3* deleted in *Prx1*+ cells (A, magnified in C, *n*=3/3). (B,D) Homozygous deletion of *Tbx3* in *Prx1*+ cells results in the absence of the lateral triceps, a hypoplastic brachialis (not visible), and presence of an ectopic muscle (B, pseudo-colored in purple, magnified in D, the long triceps is outlined in white, *n*=4/4). (E-F) At E12.5 in control and mutant forelimbs PAX7+ muscle progenitors and MYOD+ myoblasts are present where the long and lateral triceps and brachialis will develop (E,F, asterisk, magnified in insets, control *n*=3/3, mutant *n*=6/6). (G-H) *MyoD*+ myoblasts are present where the long and lateral triceps and brachialis will develop (G,H, asterisk, control *n*=5/5, mutant *n*=4/4). (I-L) No muscle fiber defects are present in *Tbx3* heterozygotes (I, magnified in K, *n*=2/2)*,* but with homozygous deletion of *Tbx3* in lateral plate mesoderm-derived cells, myofibers that will give rise to the long triceps are mis-oriented, and myofibers that presumably give rise to the lateral triceps are fewer in number and mis-oriented (J, magnified in L, *n*=2/2). (A-F,I-L) Whole-mount immunofluorescence. (A-D) Tendons are labeled using *ScxGFP*. (G,H) Whole-mount *in situ* hybridization.

We have established that TBX3 is required in the lateral plate-derived limb mesoderm for development of the lateral triceps and brachialis muscles. To determine how these muscle defects arose, we concentrated on the lateral triceps, which is superficial and more readily analyzed. We first analyzed, using whole-mount immunofluorescence, *Prx1Cre^Tg/+^*; *Tbx3^fl/+^* controls and *Prx1Cre^Tg/+^*; *Tbx3^Δ/fl^* mutants at E14.5, when the basic muscle pattern is complete (Kardon et al., 2003). By E14.5 in mutants the lateral triceps is already absent and the misaligned myofibers of the ectopic muscle are present (Fig. 7B,D compared with A,C). At E12.5 the pattern of PAX7+ progenitors and *MyoD*+ myoblasts in mutants looks similar to controls (Fig. 7E-H), indicating that the progenitors have correctly migrated into the limb and differentiated into myoblasts. We also assayed cell proliferation and apoptosis by EdU labeling and TUNEL, respectively, but found no difference between control and mutant forelimbs (data not shown). However, analysis of Myosin+ myofibers at E12.5 reveals that in mutants there is no distinct region of lateral triceps myofibers (Fig. 7I-L). In addition, misaligned myofibers are present superficially above the long head of the triceps, and these myofibers will subsequently develop into the superficial, ectopic muscle (Fig. 7K-L). Therefore TBX3 in the limb mesoderm does not regulate myogenic progenitor migration or their differentiation into myoblasts, but instead controls the formation and alignment of myofibers that become the lateral triceps.

### An individual with UMS shows bone eminence and muscle abnormalities in addition to bone defects

**Fig. 8.**
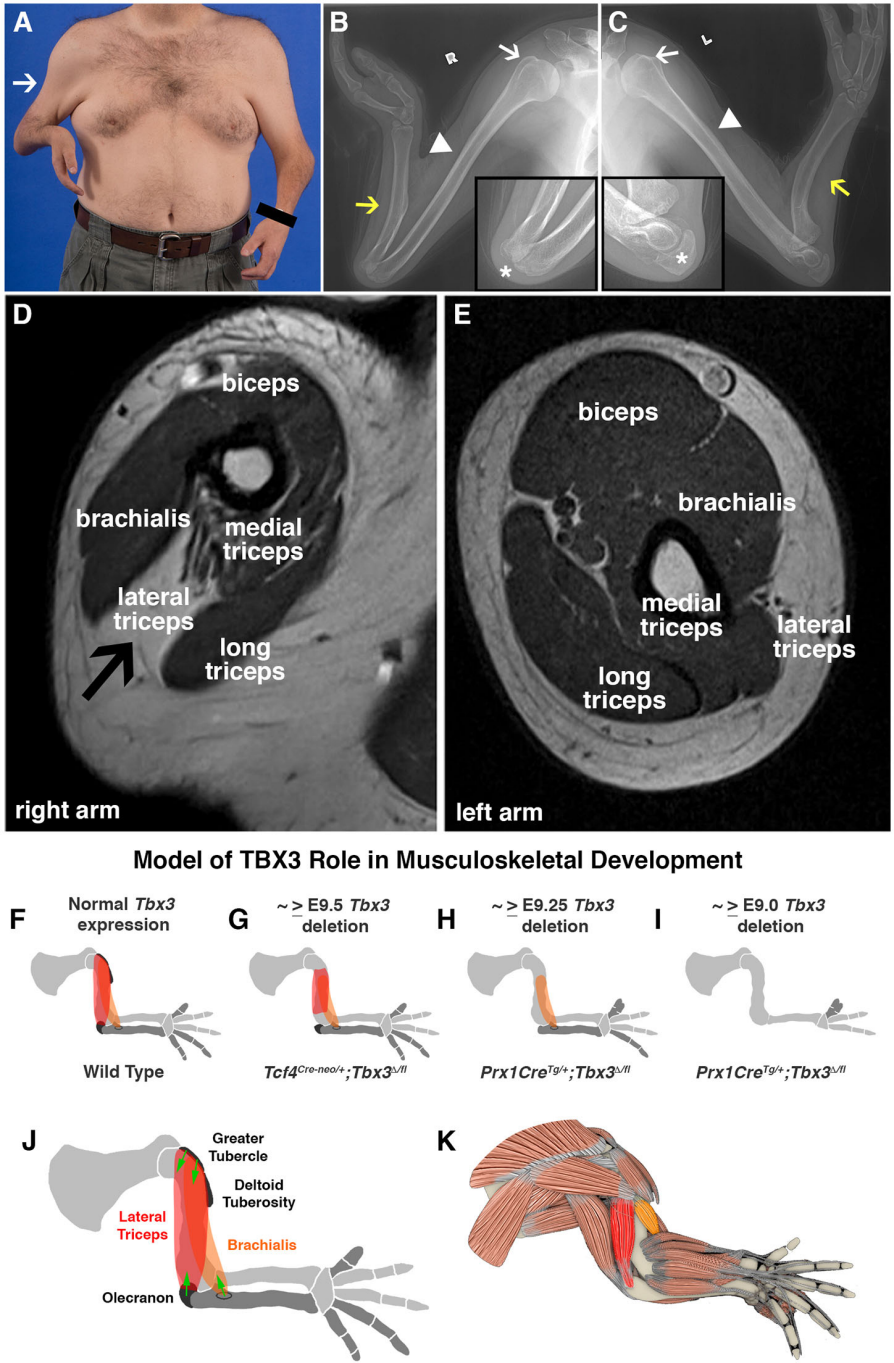
**Bone, olecranon and muscle defects are present in arms of a person with UMS.** (A) Surface anatomy of a male with UMS showing digit defects and marked concavity on the lateral surface of his right arm (arrow). (B,C) X-ray images of right (B) and left (C) arms showing bilateral loss of the ulna (yellow arrows). The greater tubercle (white arrows) and deltoid tuberosity (arrowheads) are present bilaterally. Olecranon is present on the left (C, inset) but absent on the right arm (B, inset, asterisk). (D-E) MRI images at mid-shaft humerus. The lateral triceps is absent on the right (D, arrow). All upper arm muscles are present on the left (E). (F-K) Model of the role of TBX3 in forelimb musculoskeletal development. Normal positioning of the lateral triceps (red) and brachialis (orange) in a wild-type embryo (F). Loss of muscle attachment points results in muscle development defects. Loss of one attachment point results in truncation of the muscle at that site (G,H). Loss of both attachment points results in an absent muscle (H,I). TBX3 functions non-cell-autonomously to regulate the development of the lateral triceps and brachialis (J). Anatomical location of lateral triceps and brachialis muscles (K).

Our analysis of mouse mutants shows that *Tbx3* mutations not only lead to defects in the bones, but also abnormalities in bone eminences and muscles in the forelimb. This suggested that individuals with UMS might have similar previously unrecognized phenotypic characteristics. To test this, we reassessed an individual with UMS (with a L142P *TBX3* mutation) whose forelimb skeletal defects had been previously characterized (Fig. 1 in Bamshad et al., 1999). Surface anatomy images show digit abnormalities in both arms and also a marked concavity in the right upper arm, suggestive of an underlying muscle defect (Fig. 8A). Using radiography, he was confirmed to be missing the ulna in both arms. In addition, although the greater tubercle and deltoid tuberosity seem normal, the olecranon was absent in the right arm (Fig. 8B,C). Using magnetic resonance imaging, we also identified that although all upper arm muscles are present in the left arm, the lateral triceps is absent in the right arm (corresponding to the surface anatomy concavity) and replaced by connective tissue (Fig. 8D,E). Unlike the *Tbx3* mouse mutants, the brachialis seemed normal in both of his arms. However, in humans the brachialis originates on the mid-shaft of the humerus (similar to the origin of the brachialis in the *Tbx3* mutant) and so the normal morphology of this muscle in this man is not surprising. In summary, our reanalysis of an individual with UMS demonstrates that *TBX3* has a conserved and newly expanded function in mice and humans to regulate not only the development of posterior forelimb bones but also a similar, although not identical, subset of bone eminences and muscles.

## DISCUSSION

Mutations in *TBX3* underlie UMS in humans and its distinctive defects in posterior forelimb bones. Here we show, using conditional mutagenesis in mice, that TBX3 has a broader role in forelimb development and regulates three components of the limb musculoskeleton; bones, bone eminences and muscles. Re-examination of a person with UMS shows similar previously unrecognized muscle and bone eminence defects. Our analysis of TBX3 not only increases our understanding of the phenotype and etiology of UMS, but also gives us general insights into mechanisms regulating musculoskeletal development.

Conditional deletion of *Tbx3* in the lateral plate, using *Prx1Cre*, confirms that TBX3 is required for the development of the ulna, posterior digits and the thumb (Fig. 8I) (Davenport et al., 2003; Emechebe et al., 2016; Frank et al., 2013). Because Tbx3 is expressed in the ulna and digits 4 and 5 (as revealed by lineage analysis of Tbx3+ cells) and *Prx1Cre* recombines in all limb bones, this suggests that TBX3 cell-autonomously regulates posterior bone development in the forelimb and explains why only these bones are affected by *Tbx3* loss-of-function. Thus, these data indicate that TBX3 functions differently than HOXA11 and D11, which regulate bone development non-cell-autonomously (Swinehart et al., 2013). Because *Prx1Cre* recombines in other lateral plate-derived mesoderm besides bone, future experiments specifically deleting *Tbx3* in the chondrocytes (using *Sox9Cre*; Akiyama et al., 2005) will be required to formally test whether TBX3 functions cell-autonomously. Interestingly, we have also found that severity of the bone phenotype is variable in *Prx1Cre^Tg/+^*; *Tbx3^Δ/fl^* mutants. We have interpreted the more severe phenotype (i.e. loss of ulna) to result from earlier *Prx1Cre*-mediated recombination in some embryos, as we have seen variability in timing of recombination with this transgene (Merrell et al., 2015). This suggests that specification of the ulna requires early TBX3 expression (E9.0-9.25). An alternative interpretation is that there might be slight differences in efficiency and/or extent of *Prx1Cre*-mediated recombination.

In addition to its role in regulating bones, we have identified a new role for TBX3 in regulation of a subset of eminences; the greater tubercle of the humerus, deltoid tuberosity, and olecranon (Fig. 8F-I). These eminences lie in the forelimb region where TBX3 is expressed. Thus, unlike other known regulators of eminences (e.g. TGFβ and BMP4), TBX3 is required only for this subset of eminences, and TBX3 regulation of each of these eminences differs. The different phenotypes when *Tbx3* is deleted using *Prx1Cre* or *Tcf4^Cre-neo^* suggests that the timing of TBX3 function differs between the eminences; the olecranon requires TBX3 for an early, brief period (E9.0-9.25), whereas the greater tubercle and deltoid tuberosity require TBX3 later (from E9.5) (Fig. 8F-I; Table S1). Alternatively, the different phenotypes might reflect differences in the amount or distribution of TBX3 required for each of the eminences. Also, although TBX3 is required for specification of the SOX9+ progenitors of the deltoid tuberosity, TBX3 is required for the differentiation of SOX9+ progenitors to COL2+ chondrocytes in the greater tubercle. In the olecranon, TBX3 seems to partially regulate specification, because in *Tbx3* mutants fewer SOX9+ progenitors are present, but they all appear to differentiate into COL2+ chondrocytes. Overall, the loss of eminence development with *Tbx3* deletion is similar to the phenotype of *Prx1Cre^Tg/+^*; *Bmp4^fl/fl^* loss-of-function mutants (Blitz et al., 2009) and suggests BMP4 and TBX3 might act in a common pathway in this region of the limb.

The most novel finding of our study is that TBX3 is required for development of two specific neighboring muscles, the lateral triceps and the brachialis (Fig. 8F-K). These muscles are located at the posterior margin of the dorsal-ventral boundary of the upper arm where TBX3 is expressed in lateral plate-derived limb mesoderm, and TBX3 is required non-cell-autonomously for development of these two muscles. Similar to TBX3 regulation of bone eminence formation, the timing of TBX3 regulation of the two muscles differs (Fig. 8F-I; Table S1). Development of the lateral triceps requires TBX3 during ∼E9.0-9.5, whereas the brachialis requires TBX3 for a brief period at ∼E9.0-9.25. Intriguingly, the phenotypes of the muscles are tightly correlated with the phenotypes of the bone eminences to which they attach (Fig. 8F-I; Table S1). If both origin and insertion eminences are absent, these muscles do not develop (Fig. 8I), and if only the origin or insertion is present, the muscle develops but is truncated adjacent to the missing site of attachment (Fig. 8G,H). The individual with UMS we examined has a similar, although not identical, phenotype; in the left limb the lateral triceps and its associated olecranon attachment site are missing (in humans the muscle originates on the humeral shaft). This suggests that *TBX3* has a conserved role in at least the development of the lateral triceps and the olecranon.

Our work suggests a newly discovered mechanism for specification of anatomical muscles, whereby TBX3 specifies two muscles (Fig. 8F). Our data suggest that TBX3 functions cell-autonomously in the lateral plate-derived limb mesoderm to establish the origin and insertion sites, and these in turn determine whether the lateral triceps and brachialis develop. Alternatively, TBX3 in the lateral plate mesoderm might simultaneously non-cell-autonomously specify these two muscles and cell-autonomously specify their bone attachment points. The specificity of TBX3 for these two muscles differs from other known molecular regulators of limb muscles. PAX3, LBX and SHH regulate migration of muscle progenitors (Brohmann et al., 2000; Gross et al., 2000; Hu et al., 2012; Relaix et al., 2006). Other transcription factors, such as TBX4 and TBX5, SHOX2 and HOX11 regulate the splitting of muscle masses into anatomical muscles (Hasson et al., 2010; Swinehart et al., 2013; Vickerman et al., 2011). LMX1B non-cell-autonomously specifies the dorsal identity of distal limb muscles (Li et al., 2010) and SHH non-cell-autonomously regulates anterior-posterior patterning. To our knowledge, *TBX3* is the first gene required for development of a specific subset of muscles. Surprisingly, the muscles specified by TBX3 do not correspond to any obvious anatomical compartments; the lateral triceps is an extensor muscle innervated by the radial nerve, whereas the brachialis is a flexor muscle innervated by the musculocutaneous nerve. Nevertheless, the muscles lie adjacent to one another and develop from a common muscle mass.

In summary, we demonstrate that TBX3 has a broader function in limb musculoskeletal development and UMS than previously recognized. In addition to its crucial role for development of the ulna and posterior digits, TBX3 expressed in the lateral plate is required for development of two specific muscles and their associated bone eminence attachment sites. TBX3 cell-autonomously promotes the specification and differentiation of eminence progenitors and non-cell-autonomously regulates the differentiation of lateral triceps and brachialis myofibers. The molecular identity and function of the signals downstream of TBX3 are not known and the subject of future research. Our finding that TBX3 specifies the development of a subset of muscles suggests the intriguing hypothesis that the pattern of over 40 muscles and their associated tendons and attachment sites might be specified by a collection of transcription factors expressed in different domains of the lateral plate-derived limb mesoderm.

## MATERIALS AND METHODS

### Mice

All mice lines have been previously published. We used *Tbx3^mercremer^* (Emechebe et al., 2016), *Prx1^Cre^* (Logan et al., 2002), *Pax3^Cre^* (Engleka et al., 2005), *Tcf4^GFPCre-neo^* (Mathew et al., 2011), and *Hprt^Cre^* (Tang et al., 2002); *Rosa^LacZ^* (Soriano, 1999) and *Rosa^tdTomato^* (Madisen et al., 2010) Cre-responsive reporters; *ScxGFP* (Pryce et al., 2007) tendon reporter; and *Tbx3^fl^* conditional allele (Frank et al., 2012, 2013). *Tbx3^Δ/+^* mice were generated by breeding *Tbx3^fl/+^* mice to *Hprt^Cre^* mice. Mice were back-crossed onto a C57/Bl6J background. No statistical method was used to predetermine sample size; all animals were included and the experiments were not randomized. Adult females were administered 5 mg tamoxifen in corn oil by oral gavage at indicated time point. Animal experiments were performed in accordance with protocols approved by the Institutional Animal Care and Use Committee at the University of Utah.

### Section immunofluorescence

Embryos were fixed overnight in 4% paraformaldehyde (PFA) at 4°C and either embedded in OCT or paraffin, sectioned at 12 μm or 7 μm, respectively, and immunostained. OCT sections were washed in PBS and, if required, antigen retrieved (2100 Retriever, Aptum Biologics Ltd.), using 10 mM citrate buffer (pH 6), incubated in 5% serum at room temperature (RT), and then overnight at 4°C with primary antibodies (Table S2). Sections were washed in PBS, incubated with secondary fluorescent antibodies (used at 1-5 μg/ml; Jackson Laboratories or Thermo Fisher) for 2 h at RT, washed in PBS, post-fixed in 4% PFA, rinsed in water, and mounted with Fluoromount-G (Southern Biotech). Paraffin sections were immunostained for SOX9 and COL2 (Table S2) according to Blitz et al. (2013).

### Whole-mount immunostaining and *in situ* hybridization

Embryos were fixed for 24 h in 4% PFA at 4°C, dissected, incubated for 4-24 h in Dent's bleach (1:2 30% H_2_O_2_:Dent's fix), and stored in Dent's fix (1:4 DMSO:methanol) for 2-4 weeks at 4°C. E14.5 limbs were skinned and returned to Dent's fix for 3 days prior to staining. Embryos were washed in PBS, blocked 1 h in 5% serum+20% DMSO, incubated in primary antibodies (see Table S2) for 48-72 h, washed in PBS, incubated in secondary antibodies (used at 1-5 μg/ml; Jackson Laboratories or Thermo Fisher) for 48-72 h, washed in PBS, and limbs were cleared in BA:BB (33% benzyl alcohol, 66% benzyl benzoate), all at RT. For embryos labeled with AP-conjugated anti-myosin (Table S2), embryos were heat-inactivated at 65°C for 1 h, incubated with primary antibody for 72 h, and detected with 250 μg/ml NBT and 125 μg/ml BCIP (Sigma) in alkaline phosphatase buffer. Whole-mount *in situ* hybridization was performed according to Riddle et al. (1993). Mouse *Myod* RNA probe was described previously (Brent et al., 2003).

### Skeletal staining

E14.5 embryos were fixed for 24 h in 4% PFA, rinsed with ddH_2_O for 2 days, incubated in Alcian Blue staining solution (70% EtOH, 30% glacial acetic acid, 0.2 mg/ml Alcian Blue) for 24 h at 37°C, rinsed in 100% ethanol for 3×10 min, and cleared in BABB. P0 pups were immersed in 65°C water for 1 min and skin, thoracic and abdominal organs, and fat pads were removed, fixed in 100% ethanol for 4 days, incubated in Alcian Blue staining solution [76% ethanol, 20% glacial acetic acid, 4% dH_2_O, 0.15 mg/ml Alcian Blue (Acros Organics)] for 2 days at 37°C, washed in 95% ethanol for 2×90 min, incubated in 1% KOH at RT for 5 h without rocking, and incubated in Alizarin Red staining solution [2% KOH in dH_2_O, 0.05 mg/ml Alizarin Red (Amresco)] for 1 h. Skeletal preps were cleared by incubation in 20% glycerol, 1% KOH for 6 days, then 50% glycerol, 1% KOH for 10 days, and then stored in 100% glycerol.

### β-galactosidase staining

Limbs were skinned and fixed either 4 h in 4% PFA+2 mM MgCl_2_ or overnight in 1% PFA+2 mM MgCl_2_ at 4°C, washed in PBS and rinse buffer (100 mM sodium phosphate, 2 mM MgCl_2_, 0.01% Na deoxycholate, 0.02% Igepal), and stained overnight at 37°C in X-gal staining solution (5 mM potassium ferricyanide, 5 mM potassium ferrocyanide, 1 mg/ml X-gal, rinse buffer), washed in rinse buffer, PBS, and post-fixed in 1% PFA.

### Microscopy

Fluorescence images were taken on a Nikon A1 confocal microscope. Optical stacks of whole-mount images were rendered using FluoRender (Wan, 2009). β-galactosidase and AP-conjugated anti-My32-stained embryos were imaged with a Qimaging camera.

### Magnetic resonance imaging and radiograph testing of individual with UMS

The individual with UMS was evaluated at a NIH clinical center under a protocol approved by the institutional review board of the National Institute of Neurological Disorders and Stroke. Written informed consent was obtained from the patient for all photos and videos. Muscle MRI was performed using conventional T1 weighted spin echo on a 1.5-T Achieva Phillips system. Non-contrast images were obtained from arms in the axial plane. Slices were 6 mm thick, and gaps between slices varied from 5 to 10 mm dependent on the site. Conventional radiographs of the shoulders, arms, elbows, wrists and digits were obtained with lateral and oblique views. MRI images were viewed using Osirix.
